# Morphometric analysis of maxillary air sinus: A cross sectional cadaveric study among North Indian population

**DOI:** 10.6026/973206300222611

**Published:** 2026-04-30

**Authors:** Vibhash Kumar Vaidya, Archana Chaudhary, Anand Kumar Mishra, Akhil Sathyan, Sudhakar Viswas

**Affiliations:** 1 Department of Anatomy, Veer Chandra Singh Garhwali Govt. Institute of Medical Science & Research, Srinagar, Pauri Garhwal, Uttarakhand, India; 2 Department of Anatomy, Umanath Singh Autonomous Medical College, Jaunpur, Uttar Pradesh, India; 3 Department of Anatomy, United Institute of Medical Sciences, Prayagraj, Uttar, India; 4 Department of Anatomy, Varun Arjun Medical College & Rohilkhand Hospital, Shahjahanpur, Uttar Pradesh, India; 5 Department of Physiotherapy, Jyotirao Phule Subharti College of Physiotherapy, SVSU, Meerut, Uttar Pradesh, India

**Keywords:** Maxillary sinus, morphometric analysis, cadaveric study, paranasal sinus anatomy, North Indian population

## Abstract

The anatomical dimensions of the maxillary sinus may vary across populations, with limited cadaveric data available for the North Indian population. Therefore, it is of interest to evaluate the morphometric dimensions of the maxillary sinus in North Indian cadavers, assessing bilateral variations. The results revealed the mean sinus height, width, anteroposterior length and volume in this population. Slight variations were noted between sides, but the differences were not statistically significant. Thus, we report baseline morphometric data of the maxillary sinus in the North Indian population to aid safer surgical planning.

## Background:

The maxillary air sinus, also known as the Maxillary sinus, is the largest of the paranasal sinuses and plays a crucial role in craniofacial anatomy. It is a pyramidal-shaped cavity located within the body of the maxilla, adjacent to the nasal cavity, orbit and oral cavity. The maxillary sinus is lined by a thin respiratory mucosa known as the Schneiderian membrane and communicates with the nasal cavity through the ostium in the middle meatus [1]. Anatomically, it is bounded superiorly by the orbital floor, medially by the lateral wall of the nasal cavity, anteriorly by the facial surface of the maxilla, posteriorly by the infratemporal surface and inferiorly by the alveolar process of the maxilla. Because of its close relationship with vital structures such as the teeth, nasal cavity and orbit, the maxillary sinus has significant clinical, surgical and forensic importance. Morphometric analysis of the maxillary sinus refers to the quantitative measurement of its dimensions, including height, width, length and volume [2]. These measurements provide valuable information about anatomical variations among different populations and individuals. The size and shape of the maxillary sinus may vary according to age, sex, genetic factors and environmental influences. Such variations are important in understanding craniofacial development and are also helpful in anthropological and forensic identification. Morphometric studies contribute to establishing baseline anatomical data that can assist clinicians and surgeons in planning procedures involving the maxillary region [3]. The maxillary sinus begins its development during the early fetal period as an evagination of the lateral wall of the nasal cavity. After birth, it gradually expands through a process known as pneumatization, reaching its maximum size during adulthood [4]. During growth and development, the sinus enlarges in close relation to the eruption of teeth and the development of surrounding facial structures. Loss of teeth and aging may further influence sinus dimensions due to increased pneumatization of the maxilla. Because of these developmental changes, the morphometry of the maxillary sinus can differ significantly across individuals and populations. In clinical practice, the anatomy of the maxillary sinus is highly relevant in several dental and maxillofacial procedures [5]. Surgical interventions such as sinus lift procedures, dental implant placement, endodontic surgery and maxillofacial trauma management require precise knowledge of sinus dimensions and anatomical relationships. Improper understanding of sinus morphology can lead to complications such as sinus membrane perforation, infection, oroantral fistula formation and implant failure. Therefore, accurate morphometric data are essential for safe surgical planning and execution. Morphometric evaluation of the maxillary sinus also plays an important role in radiology and diagnostic imaging [6].

Modern imaging techniques such as computed tomography (CT) and cone beam computed tomography (CBCT) have significantly improved the ability to visualize and measure sinus dimensions in living individuals. However, cadaveric studies remain a gold standard for obtaining precise anatomical measurements, as they allow direct observation and measurement of the sinus without radiographic distortion. Cadaver- based morphometric research provides detailed insights into anatomical variations that may not always be evident through imaging studies alone. Apart from clinical relevance, the maxillary sinus has considerable importance in forensic science and anthropology [7]. The morphology and dimensions of the sinus can be used as a reliable parameter for identification of sex and estimation of population characteristics. Since the maxillary sinus remains intact even when other skeletal structures are damaged, it serves as a useful anatomical landmark in forensic investigations involving skeletal remains. Population-specific morphometric data help forensic experts develop reference standards that can aid in identification processes. Several studies have reported variations in the size and shape of the maxillary sinus among different ethnic and geographic populations [8]. Factors such as genetic background, environmental conditions and dietary habits may influence craniofacial morphology, including sinus development. However, there is limited cadaveric data available regarding the morphometric characteristics of the maxillary sinus in the North Indian population. Considering the diversity in craniofacial features among different regional groups in India, it is essential to establish region-specific anatomical data through detailed morphometric studies. Cadaveric studies offer a unique opportunity to obtain accurate measurements and analyze structural variations directly [9]. These studies provide valuable anatomical references for clinicians, anatomists and researchers. Establishing morphometric parameters specific to the North Indian population can contribute to better understanding of anatomical variations and improve clinical outcomes in dental and maxillofacial procedures [10]. Therefore, it is of interest to report the morphometric dimensions of the maxillary air sinus in cadavers of the North Indian population to provide baseline anatomical data that may assist clinicians, surgeons and researchers in improving diagnostic accuracy, surgical planning and forensic identification.

## Methodology:

## Study design and setting:

This study was designed as a cross-sectional cadaveric study conducted in the Department of Anatomy VCSG Government Institute of Medical Science & Research, Srinagar, Pauri Garhwal, Uttarakhand, Umanath Singh Autonomous Medical College, Jaunpur, & United Institute of Medical Sciences, Prayagraj, Uttar Pradesh. The study aimed to evaluate the morphometric dimensions of the Maxillary sinus in adult human cadavers. Ethical approval for the study was obtained from the Institutional Ethics Committee prior to the commencement of the research. All procedures were performed in accordance with ethical standards for research involving human cadaveric specimens.

## Study sample:

A total of 100 maxillary sinuses obtained from 50 adult cadavers were included in the study. Both the right and left maxillary sinuses of each cadaver were examined, giving a total of 100 sinus cavities for morphometric analysis. The cadavers used in this study were preserved specimens available in the Department of Anatomy and belonged to individuals from the North Indian population.

## Inclusion criteria:

Cadavers were included in the study based on the following criteria:

[1] Adult cadavers aged above 18 years.

[2] Cadavers with intact maxilla and paranasal sinus region.

[3] Cadavers without visible deformities or damage to the maxillary region.

[4] Cadavers belonging to the North Indian population as recorded in institutional records.

## Exclusion criteria:

Cadavers were excluded from the study if they met any of the following conditions:

[1] Evidence of trauma, fractures, or surgical intervention in the maxillary region.

[2] Presence of pathological lesions, tumors, or congenital abnormalities affecting the maxillary sinus.

[3] Poorly preserved cadavers or those with damaged sinus walls that could affect accurate measurement.

## Dissection procedure:

The dissection of the maxillary sinus was performed following standard anatomical procedures. The anterior wall of the maxilla was carefully exposed by removing the overlying soft tissues. Using bone cutting instruments, the anterior wall of the maxilla was opened cautiously to expose the maxillary sinus cavity without disturbing its internal boundaries. The mucosal lining was gently removed to clearly visualize the sinus cavity and its anatomical limits.

## Morphometric measurements:

Morphometric measurements of the maxillary sinus were taken using a digital vernier caliper with an accuracy of 0.01 mm and a precision scale. The measurements were recorded for various dimensions, including the antero-posterior (front to back), medial-lateral (side to side), and superior-inferior (top to bottom) lengths of the maxillary sinus. In addition to these linear dimensions, the volume of the maxillary sinus was estimated using a standard geometric formula based on the measured dimensions. The data obtained from these morphometric measurements were then analyzed to assess the variations in the sinus size across different subjects and correlate them with other anatomical features of the maxillary region. These measurements provide critical information for clinical applications such as sinus surgery, dental implant placement, and understanding the variability in the anatomy of the maxillary sinus across populations.

## Measuring scale:

The following parameters were recorded:

[1] Maximum height - measured from the lowest point of the sinus floor to the highest point of the sinus roof.

[2] Maximum width - measured from the medial wall to the lateral wall of the sinus.

[3] Maximum anteroposterior length - measured from the anterior wall to the posterior wall of the sinus.

[4] Sinus volume (estimated) - calculated using standard geometric approximation formulas based on recorded dimensions.

All measurements were taken bilaterally for each cadaver. To minimize measurement error, each parameter was measured three times and the average value was recorded as the final measurement. In Figure 1 (see PDF), the morphometric measurements of the maxillary sinus are illustrated, showing the key parameters such as maximum height, maximum width, maximum anteroposterior length, and estimated sinus volume. These dimensions were recorded using a digital vernier caliper and calculated based on standard geometric formulas to assess the anatomical variability of the maxillary sinus.

## Data collection:

All morphometric data were systematically recorded in a pre-designed data collection sheet. The right and left maxillary sinus measurements were documented separately. Additional demographic details available in cadaver records, such as age and sex, were also noted when available.

## Statistical analysis:

The collected data were entered into Microsoft Excel and analyzed using Statistical Package for Social Sciences (SPSS) version 25.0. Descriptive statistics such as mean, standard deviation, minimum and maximum values were calculated for all morphometric parameters. Comparisons between the right and left maxillary sinuses were performed using the paired t-test. A p-value < 0.05 was considered statistically significant.

## Outcome measures:

The primary outcome of the study was to determine the mean morphometric dimensions of the maxillary sinus in the North Indian cadaveric population. Secondary outcomes included comparison of measurements between the right and left sides and assessment of anatomical variations in sinus dimensions. This methodological approach enabled accurate morphometric evaluation of the maxillary sinus and provided reliable baseline anatomical data for the North Indian population.

## Results:

The study aimed to evaluate the morphometric dimensions of the maxillary sinus in the North Indian population. The mean measurements for the maxillary sinus were as follows: the maximum height was found to be 33.84 ± 3.92 mm, the maximum width was 24.67 ± 3.15 mm and the maximum anteroposterior length was 36.12 ± 4.05 mm. additionally, the estimated sinus volume was 15.32 ± 2.87 cm^3^. These findings are summarized in Table 1 (see PDF), which provides the mean values and standard deviations for each morphometric parameter. The study also compared the measurements between the right and left maxillary sinuses. Slightly higher dimensions were observed on the right side compared to the left, but the differences were not statistically significant (p > 0.05), as shown in Table 2 (see PDF). This suggests that while there were some variations between the sides, they were not substantial enough to affect clinical practice significantly. Further analysis of the bilateral symmetry of the maxillary sinuses demonstrated overall symmetry in the dimensions, with no statistically significant differences between the right and left sides for any of the parameters measured, including height, width and anteroposterior length. This comparison is presented in Table 3 (see PDF). These findings indicate moderate variation in maxillary sinus dimensions, but with overall bilateral symmetry in the North Indian population. The results provide important baseline anatomical data for clinicians involved in maxillofacial surgeries, dental implant placement and sinus lift procedures. The data from this study could help improve the safety and efficacy of maxillofacial procedures by offering a detailed understanding of the maxillary sinus morphology in this population (Table 4 - see PDF).

## Discussion:

The present cross-sectional cadaveric study evaluated the morphometric dimensions of the Maxillary sinus in the North Indian population. Accurate knowledge of maxillary sinus morphology is important for various dental and maxillofacial procedures such as implant placement, sinus lift surgery and endodontic surgery. Anatomical variations in sinus dimensions may influence surgical outcomes; therefore, population- specific morphometric data are essential for clinical planning. In the present study, the mean height, width and anteroposterior length of the maxillary sinus showed moderate variability, with slight predominance of measurements on the right side compared to the left side. However, the differences between the two sides were statistically insignificant. These findings suggest that the maxillary sinus generally maintains bilateral symmetry in most individuals. The results of this study provide baseline morphometric data for the North Indian population, which may help clinicians in planning surgical interventions involving the maxillary region. The findings of the present study are comparable with the study conducted by Uchida *et al.* (1998) [11] who performed a cadaveric investigation to determine the dimensions and volume of the maxillary sinus. Their study reported a mean anteroposterior length of 30.1 mm, height of 34.6 mm, width of 25.4 mm and mean sinus volume of approximately 11.3 cm^3^. These values are relatively close to those observed in the present study, although slight differences may be attributed to variations in ethnicity, sample size and measurement techniques. The authors also emphasized that knowledge of sinus dimensions is crucial for determining the required bone graft volume during sinus floor augmentation procedures. Similarly, Gosau *et al.* (2009) [12] conducted a cadaveric study evaluating the anatomy and volume of the maxillary sinus. Their investigation highlighted the considerable anatomical variability of the sinus and emphasized its clinical relevance in maxillofacial surgery. The authors reported variations in sinus volume and anatomical landmarks such as the semilunar hiatus and sinus septa, indicating that detailed anatomical understanding is essential to prevent surgical complications.

These findings support the observations of the present study that the maxillary sinus demonstrates measurable variability among individuals. Another cadaveric investigation by Rosano *et al.* (2010) [13] focused on anatomical variations within the maxillary sinus, particularly the presence and location of antral septa. The authors reported that septa were present in approximately 40% of specimens, with a mean height of 8.72 mm and were commonly located in the region between the first and second molars. Although the primary focus of that study was septal morphology, the authors emphasized that anatomical variations in the sinus cavity must be considered during sinus lift procedures to prevent membrane perforation. The current study also highlights the importance of understanding sinus morphology for safe surgical intervention. More recently, Panchal *et al.* (2025) [14] performed a three-dimensional morphometric and volumetric analysis of the maxillary sinus using cone-beam computed tomography in an Indian population. Their results showed that the mean anteroposterior diameter ranged from 36-38 mm, while the craniocaudal height was approximately 38-40 mm, with slight side-to-side variations. These measurements are broadly consistent with the present cadaveric findings, suggesting that sinus morphometry in Indian populations may follow similar patterns. The authors also reported minor right-left differences, which aligns with the minimal asymmetry observed in our study.

A large CBCT-based morphometric study conducted by Alshammari *et al.* (2026) [15] in a Saudi population reported that maxillary sinus dimensions vary according to sex, with males demonstrating significantly larger sinus measurements compared to females. The authors also noted slight right-left asymmetry, although the difference was clinically insignificant. These findings correspond with the results of the present study, which also observed minor side-wise differences but no statistically significant variation between the right and left sinuses. Overall, the present study is consistent with previous research indicating that the maxillary sinus exhibits moderate anatomical variability but generally maintains bilateral symmetry. Differences observed between studies may be explained by variations in methodology, population characteristics, imaging techniques and sample size. Cadaveric studies, such as the present investigation, provide direct anatomical measurements and therefore offer highly reliable morphometric data. The morphometric information obtained from this study contributes to the existing literature by providing population-specific data for the North Indian population. Such data are valuable for clinicians involved in dental implantology, maxillofacial surgery and endodontic procedures. Additionally, these findings may also be useful in forensic identification and anthropological research where craniofacial structures, including the maxillary sinus, are used as anatomical markers. Overall, the present study supports the existing evidence that detailed knowledge of maxillary sinus morphology is essential for improving surgical safety, diagnostic accuracy and anatomical understanding of the paranasal sinus region.

## Conclusion:

Important morphometric data regarding the Maxillary sinus in the North Indian population is evaluated. The results showed moderate variation in sinus dimensions, with generally symmetrical measurements between the right and left sides. These data provides baseline anatomical information that may assist clinicians in dental implantology, maxillofacial surgery and endodontic procedures. Knowledge of sinus morphology can help reduce surgical complications and improve treatment planning. Therefore, detailed understanding of maxillary sinus morphometry is essential for safe and effective clinical practice.

## Limitations:

The present study has certain limitations that should be considered while interpreting the findings. First, the study was conducted on a relatively limited sample size of 50 cadavers (100 maxillary sinuses) from a single anatomical institution, which may not fully represent the entire North Indian population. Second, detailed demographic information such as exact age, sex and dental status of all cadavers was not consistently available, which may influence the morphometric dimensions of the Maxillary sinus. Third, as this was a cadaveric cross- sectional study, the findings may differ from measurements obtained through radiological methods such as CT or CBCT in living individuals due to tissue preservation changes. Additionally, the study focused only on basic linear morphometric parameters and estimated volume, while other anatomical variations such as sinus septa, mucosal thickness and ostium position were not evaluated. Therefore, further studies with larger multicentric samples and advanced imaging techniques are recommended to obtain more comprehensive morphometric data.
